# Longitudinal gut microbial signals are associated with weight loss: insights from a digital therapeutics program

**DOI:** 10.3389/fnut.2024.1363079

**Published:** 2024-07-08

**Authors:** Shreyas V. Kumbhare, Inti Pedroso, Bharat Joshi, Karthik M. Muthukumar, Santosh K. Saravanan, Carmel Irudayanathan, Gursimran S. Kochhar, Parambir S. Dulai, Ranjan Sinha, Daniel E. Almonacid

**Affiliations:** ^1^Digbi Health, Mountain View, CA, United States; ^2^Division of Gastroenterology, Hepatology and Nutrition, Allegheny Health Network, Pittsburgh, PA, United States; ^3^Division of Gastroenterology, Northwestern University, Chicago, IL, United States

**Keywords:** microbiome, obesity, weight loss, intervention, diet

## Abstract

**Introduction:**

The gut microbiome’s influence on weight management has gained significant interest for its potential to support better obesity therapeutics. Patient stratification leading to personalized nutritional intervention has shown benefits over one-size-fit-all diets. However, the efficacy and impact on the gut’s microbiome of personalizing weight loss diets based on individual factors remains under-investigated.

**Methods:**

This study assessed the impact of Digbi Health’s personalized dietary and lifestyle program on weight loss and the gut microbiome end-points in 103 individuals. Participants’ weight loss patterns and gut microbiome profiles were analyzed from baseline to follow-up samples.

**Results:**

Specific microbial genera, functional pathways, and communities associated with BMI changes and the program’s effectiveness were identified. 80% of participants achieved weight loss. Analysis of the gut microbiome identified genera and functional pathways associated with a reduction in BMI, including Akkermansia, Christensenella, Oscillospiraceae, Alistipes, and Sutterella, short-chain fatty acid production, and degradation of simple sugars like arabinose, sucrose, and melibiose. Network analysis identified a microbiome community associated with BMI, which includes multiple taxa known for associations with BMI and obesity.

**Discussion:**

The personalized dietary and lifestyle program positively impacted the gut microbiome and demonstrated significant associations between gut microbial changes and weight loss. These findings support the use of the gut microbiome as an endpoint in weight loss interventions, highlighting potential microbiome biomarkers for further research.

## Introduction

Obesity and related comorbidities, such as heart disease, type 2 diabetes, and certain cancers are a significant public health burden, leading to rising costs for healthcare systems. Despite continuous efforts in developing weight-loss strategies and public health campaigns, most interventions fail at a population level to achieve effective long-term weight management. Obesity is a complex disorder with a multifaceted etiology influenced by a complex interplay of genetic, environmental, and lifestyle factors. While genetics lay the groundwork for susceptibility, environmental influences, particularly diet and physical activity, and epigenetic modifications play pivotal roles in the manifestation of obesity (1, 2). Over the past decade, research has unveiled the critical role of the gut microbiome in the development of obesity and its contribution to the reversal of metabolic disorders in general (3–6), placing the gut microbiome at the center of new strategies to prevent, treat and reverse obesity.

The human gut microbiome is a complex and dynamic community of microorganisms essential for maintaining human health (7), and it is influenced by various factors, including diet and lifestyle (8, 9). The human gut microbiome plays a critical role in the metabolism of nutrients and other substances and can produce a wide range of beneficial and harmful metabolites (10). For example, gut bacteria can ferment dietary fibers that are indigestible by human enzymes, producing short-chain fatty acids (SCFAs) that serve as an energy source for the gut lining cells and can also influence overall body metabolism (10). Conversely, certain animal-food rich diets are associated with the production of trimethylamine (TMA). TMA is subsequently converted into trimethylamine N-oxide (TMAO) by the liver, and elevated levels of TMAO in the bloodstream have been linked to an increased risk of cardiovascular disease (11, 12). Changes in the taxonomic composition of the gut microbiome can therefore affect the balance of microbial metabolic pathways, leading to changes in the types and amounts of metabolites produced, with important implications for human health.

In recent years, there has been increasing interest in studying the longitudinal changes in the gut microbiome over time as a response to different interventions and their relationship to various health outcomes, including body mass index (BMI) (13). Research studies have shown that different diets can modulate the gut microbiome in individuals with obesity (14, 15). For example, a high-fat diet may increase the abundance of certain bacteria associated with obesity. In contrast, a low-fat diet may increase the abundance of bacteria associated with leanness (5, 16). A few reports have shown differences in the gut microbiome taxonomic composition, diversity, and metabolic pathways encoded in individuals with different BMIs (13, 17, 18), and that different dietary patterns result in distinct patterns of microbial taxa (8). Understanding the intricate association between diet and the gut microbiome is therefore crucial to deciphering the mechanisms underlying the development of various disease conditions. Although it is evident from studies that dietary interventions, such as changing the types and amounts of food that are consumed, are associated with the composition and function of the gut microbiome, it is not well understood how these differences evolve over time (13).

Recent advances in gut microbiome research have provided insights into the associations between dietary components, gut microbiome composition, and weight regulation allowing for the development of dietary interventions targeting the gut microbiome to achieve effective weight loss (17, 19, 20). A growing body of research explores the impact of dietary interventions on obesity, type 2 diabetes (T2D), and non-alcoholic fatty liver disease (NAFLD). These studies encompass various dietary approaches, including interventions enriched with sardines (21), specific fat types (22), or traditional foods like yogurt (23). Notably, Balfegó et al. (2016) investigated a T2D diet supplemented with sardines, observing decreased Firmicutes and increased *E. coli* in both disease and control groups, although only the control group saw a significant HbA1c reduction (21). This suggests that specific dietary components, like sardines in this case, may not universally translate to improved glycemic control. Other dietary interventions targeting gut microbiota have also been explored. For example, Huang et al. found that an Okinawan-based Nordic diet did not alter Enterobacteriaceae abundance, microbial diversity, or SCFAs (24). Frost et al. employed a 3-phase weight loss program, achieving weight loss and improved glycemic control in all participants (25). Prior studies often involved smaller cohorts and, crucially, standardized interventions across individuals. Digbi Health has developed a digital therapeutics program based on the participants’ multi-omic signals (genetics and gut microbiome), self-reported data, and data from wearable devices. The outcomes of the weight loss program in different cohorts of individuals have been recently published, demonstrating its applicability not only in weight loss (26), but also in reducing fasting blood glucose and HbA1c levels (27), improving mental health symptoms (28), and functional gastrointestinal disorders (29).

In this study, we aimed to investigate the effects of Digbi Health’s personalized dietary and lifestyle weight loss program on the gut microbiome composition over time and its relationship to BMI. To understand the impact of this program on the gut microbiome, we performed longitudinal microbiome sampling at the beginning and approximately at 6 months follow-up. Our analyses identified changes over time in the abundances of microbial taxa and their encoded metabolic pathways, alpha and beta diversity, and the structure of microbial co-abundance networks in response to the weight loss program.

## Methods

### Cohort enrollment and inclusion criteria

The study subjects enrolled in the Digbi Health personalized care program for weight loss, which is a commercially available product known as Digbi Control™, are all 18 years of age and above, located in the United States at the time of the study (between August 2019 to November 2021), and have access to this health program as part of their employers’ benefits system. For this retrospective observational study, the inclusion criteria were: 18 years and older (mandatory), and BMI ≥ 30, or BMI between 25 and 30 with a cardiometabolic comorbidity, or diagnosed with prediabetes or Type 2 Diabetes, NAFLD, pancreatitis. Only subjects with gut microbiome data at two-time points were included. The exclusion criteria was subjects with antibiotics consumption (self-reported) at the enrollment date. Research associated with this weight loss program has been reviewed and approved by the Institutional Review Board of E&I Review Services (protocol code #18053 on 05/22/2018). All subjects considered for the present manuscript provided their research informed consent electronically as part of their written informed consent form.

### Digital therapeutics weight loss program

The primary objective of the Digbi Health intervention program is to assist participants in achieving weight loss while addressing weight-related inflammatory disorders and associated comorbidities affecting multiple bodily systems. Digbi Health is an innovative digital therapeutics platform that leverages artificial intelligence (AI) models within a scientific framework. This framework encompasses genetic and gut microbiome profiling, baseline participant information, and a comprehensive health monitoring system through an app. Upon enrollment, program participants were provided with online login access to the Digbi Health app and were asked to complete a health questionnaire. A Bluetooth-compatible digital weighing scale, and buccal swab and stool sampling kits were shipped to all participants. The app was used to track subjects’ weight, assess dietary intake (via uploaded photographs of food items consumed), and track wellness and lifestyle-associated metrics such as sleep quality and quantity, exercise type and duration, stress and meditation, energy levels, cravings, and recommended foods consumed/avoided. Dietary intake was assessed by coaches who assigned a nutrient density score to meals based on their inflammatory, fiber diversity, and expected insulin response. The results of the analysis of genetic and gut microbiome profiles were evaluated by a health coach. Genetic profiling helps assess predisposition to diseases like obesity, chronic gastrointestinal disorders, cardiometabolic conditions, behavioral and mental health traits, as well as food allergies and intolerances. Digbi Health utilizes gut microbiome data to identify taxonomic and functional markers of health, such as anti-inflammatory compounds, beneficial keystone species, metabolic pathways, and food tolerances. This enables an evaluation of how different foods may impact the gut microbial community, supporting personalized food recommendations for weight loss and improved overall health. The multi-omics data, derived from genetic makeup and gut microbiome at multiple time points, informs the identification of diagnostic and therapeutic indicators. These indicators are then integrated with an individual’s lifestyle choices and demographic information to develop personalized dietary and lifestyle plans based on their unique multi-omics data.

Program participants were guided to make appropriate food choices based on their food preferences, lifestyle (what time of the day they eat, where they eat, whether they are cooking for themselves or the whole family, etc), food allergies or intolerances self-reported or detected through genetic testing (e.g., lactose intolerance), specific gastrointestinal symptoms (bloating, constipation or diarrhea) or other symptoms (e.g., anxiety levels, stress, sleep problems). Program participants were provided with recipes and meal plans that match their profile. To achieve its goal, the program sought to nudge participants toward making incremental lifestyle changes focused on reducing sugar consumption and timing meals to optimize insulin sensitivity, reducing systemic inflammation by identifying possibly inflammatory and anti-inflammatory nutrients, and increasing fiber diversity to improve gut health. Most importantly, these behavioral changes were implemented with the help of virtual health coaching and the app to ensure that these changes are habit-forming, i.e., long-term sustainable.

### Sample collection and processing

Subjects self-collected fecal samples using fecal swabs (Mawi Technologies iSWAB Microbiome collection kit, Model no. ISWAB-MBF-1200). Sample collection was completed by following standardized directions provided to all subjects in an instruction manual. DNA extraction was performed using Qiagen MagAttract Power Microbiome DNA Kit on an automated liquid handling DNA extraction instrument, followed by bacterial 16S rRNA gene V3-V4 region amplification and sequencing on the Illumina MiSeq platform using 2 × 300 bp paired-end sequencing performed at Akesogen Laboratories in Atlanta, GA. Sequence reads were demultiplexed, denoised, and ASVs generated using DADA2 in QIIME2 version 2021.4 (30).

### Microbiome data analysis

We collected 206 stool swab samples (103 individuals at two-time points, T1: Early phase, and T2: Follow-up phase). Initial quality control steps included the removal of primers and low-quality bases, and removing ASVs classified as non-bacterial sequences, or unassigned phyla. Taxa were agglomerated at genus levels, and those with low abundance (taxa with <10 reads in at least 10% of samples) were excluded, resulting in a reduction of the sparsity of the abundance matrix from 99.75 to 37.6% (with an average of 98.3% of read retention) and removal of singletons. The abundance matrix was rarefied (unless mentioned otherwise) at even depth (*n* = 61,000 reads per sample (minimum reads across the samples) with 500 iterations) using QIIME2 (31), resulting in 155 taxa. The abundance of functional microbial pathways related to gut and neuroactive metabolites (32) was calculated with the q2-picrust2 plugin (v2.4.2) in QIIME2 (33) and the Omixer-RPM package (version 0.3.2) (34). All raw abundances were centered-log ratio (CLR) transformed unless otherwise specified (35). Details of network modules and other statistical analyses are provided in the Supplementary material. The microbiome sequence data used in this study were submitted to NCBI SRA under Bioproject accession number PRJNA907500.

## Results

### Cohort characteristics

The study consisted of three time points, T0: baseline phase (enrollment), T1: early phase, and T2: follow-up phase. Gut microbiome samples were collected at T1 and T2. At baseline, the cohort comprised 75.7% of females with an average age of 53.55 (Median 55.0; IQR: 44.5, 63.0). Almost 60% of individuals were suffering from FGIDs (at least one self-reported functional gastrointestinal disorder: IBS, gassiness, bloating, constipation, diarrhea, or dyspepsia), while ~86% had other comorbidities in addition to overweight or obese BMIs at baseline. 35% of individuals were on prescribed antidepressants or anxiolytics, while 14.6% were using recreational drugs at baseline.

**Figure 1:**
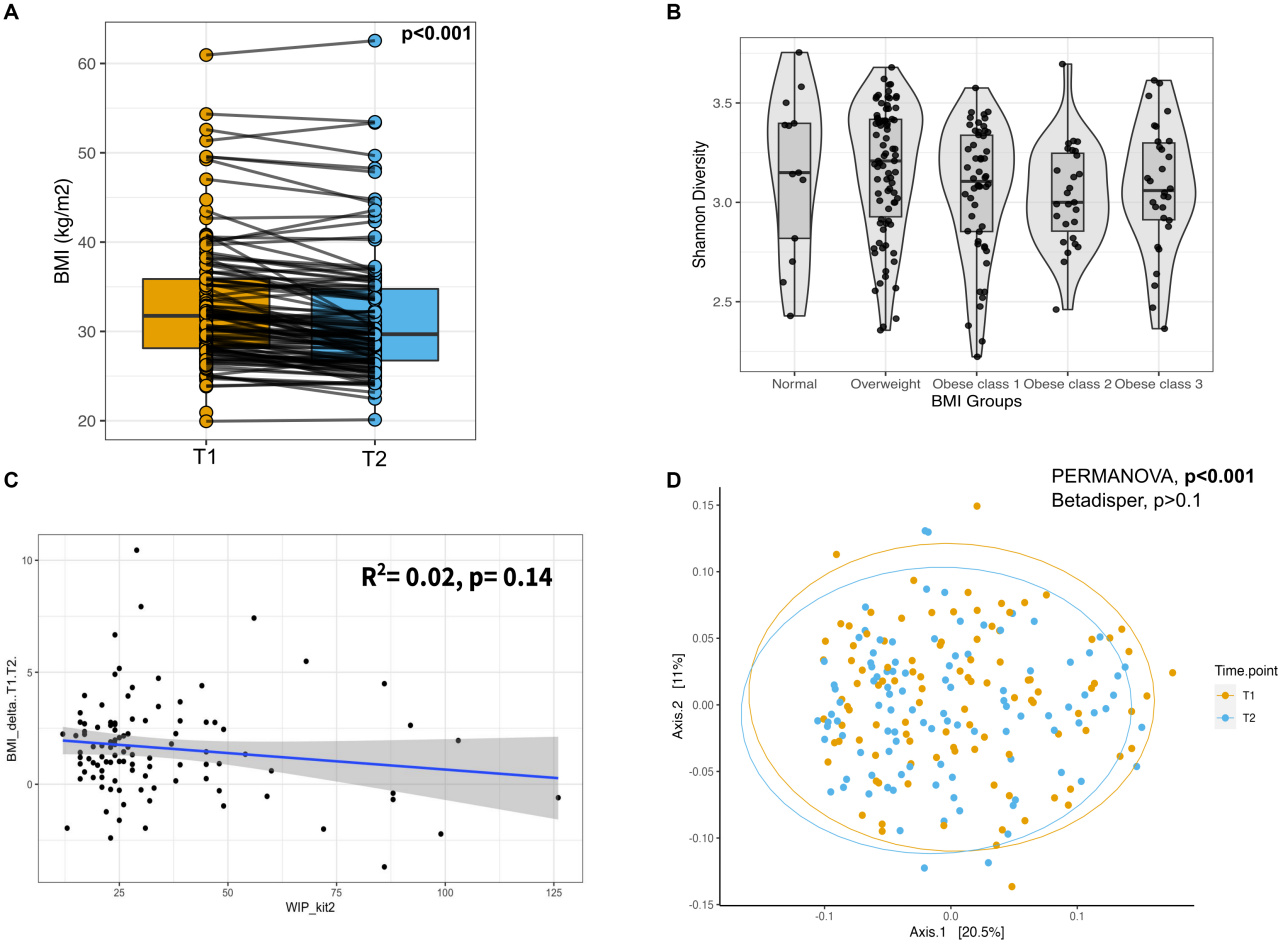
Weight loss after participating in the program and gut microbial composition. **(A)** Box-plot of BMI for the individuals at T1 and T2. Measurements of the same individual are linked with a line across the two-time points. *p*-value from Wilcoxon-signed rank test. **(B)** Box-plot displaying the Shannon diversity across different BMI groups. **(C)** Scatter plot showing the regression analysis of change in BMI (T2-T1) and Weeks in Program (WIP) until T2. **(D)** Principal Coordinate Analysis (PCoA) plot showing the beta diversity of the gut microbes across all individuals based on the Bray–Curtis dissimilarity. Ellipses represent 95% confidence regions. PERMANOVA analysis showed significant differences (shift) in overall diversity between two-time points (*p*-value <0.001). PERMANOVA, Permutational Multivariate Analysis of Variance; T1, Early phase; T2, Follow-up phase.

Most individuals, approx. 80%, lost weight with an average reduction in BMI from T0 to T2 of 2.57 BMI units (*p*-value<0.001), and from T1 to T2 of 1.6 BMI units (*p*-value<0.001; see Table 1 and Figure 1A). From T0 to T2 14.6, 34, and 28.2% of individuals lost 3–5%, 5–10, and > 10% of body weight, respectively. Furthermore, 17.5, 31.1, and 14.6% of individuals lost 3–5%, 5–10, and > 10% of body weight, respectively, from T1 to T2. Although there was variation in the program duration across the cohort until T2, it had no significant association with the amount of weight loss (*p*-value = 0.14, Figure 1C). Additionally, we did not observe any significant influence of age, gender, BMI at T0, and use of anxiolytics and antidepressants at T0, except alcohol consumption at T0 on percentage body weight loss (Supplementary Table S1).

**Table 1:** Study cohort and demographic characteristics.

Cohort Characteristic	Total, *n* = 103
Age Median (IQR)	55.0 (44.5, 63.0)
Gender *n* (%)	
Male	25 (24.3%)
Female	78 (75.7%)
Weight profile (in lbs)	
Weight at T0 (Start weight)	199.2 (175.0, 235.0)
Weight at T1	195.5 (170.3, 226.9)
Weight at T2	181.8 (162.2, 215.8)
Change in weight (T1-T2)	8.6 (2.0, 15.9)
Individuals with weight loss (at T2)	82 (79.6%)
BMI kg/m^2^ (Median, IQR)	
BMI at T0 (Start BMI)	32.3 (29.0, 37.4)
BMI at T1	31.8 (28.3, 36.6)
BMI at T2	29.7 (26.9, 34.8)
Change in BMI (T1-T2)	1.4 (0.3, 2.7)
Consume alcohol at T0 *n* (%)	66 (64.1%)
Weeks in program until T2 (Median, IQR)	26 (21.0, 39.0)
Clinical characteristics	
Used recreational drugs at T0 *n* (%)	15 (14.6%)
FGID at T0 *n* (%)	61 (59.2%)
Other comorbidities at T0 *n* (%)	88 (85.4%)
Use of antidepressants or anxiolytics at T0 *n* (%)	36 (35%)

Values are expressed as median (IQR) and percentages of available data. FGID, Functional Gastro-Intestinal Disorders; BMI, Body Mass Index; T0, at Baseline (during enrollment); T1, Early phase; T2, Follow-up phase.

### Body mass index is associated with alpha and beta gut microbial diversity

**Figure 2:**
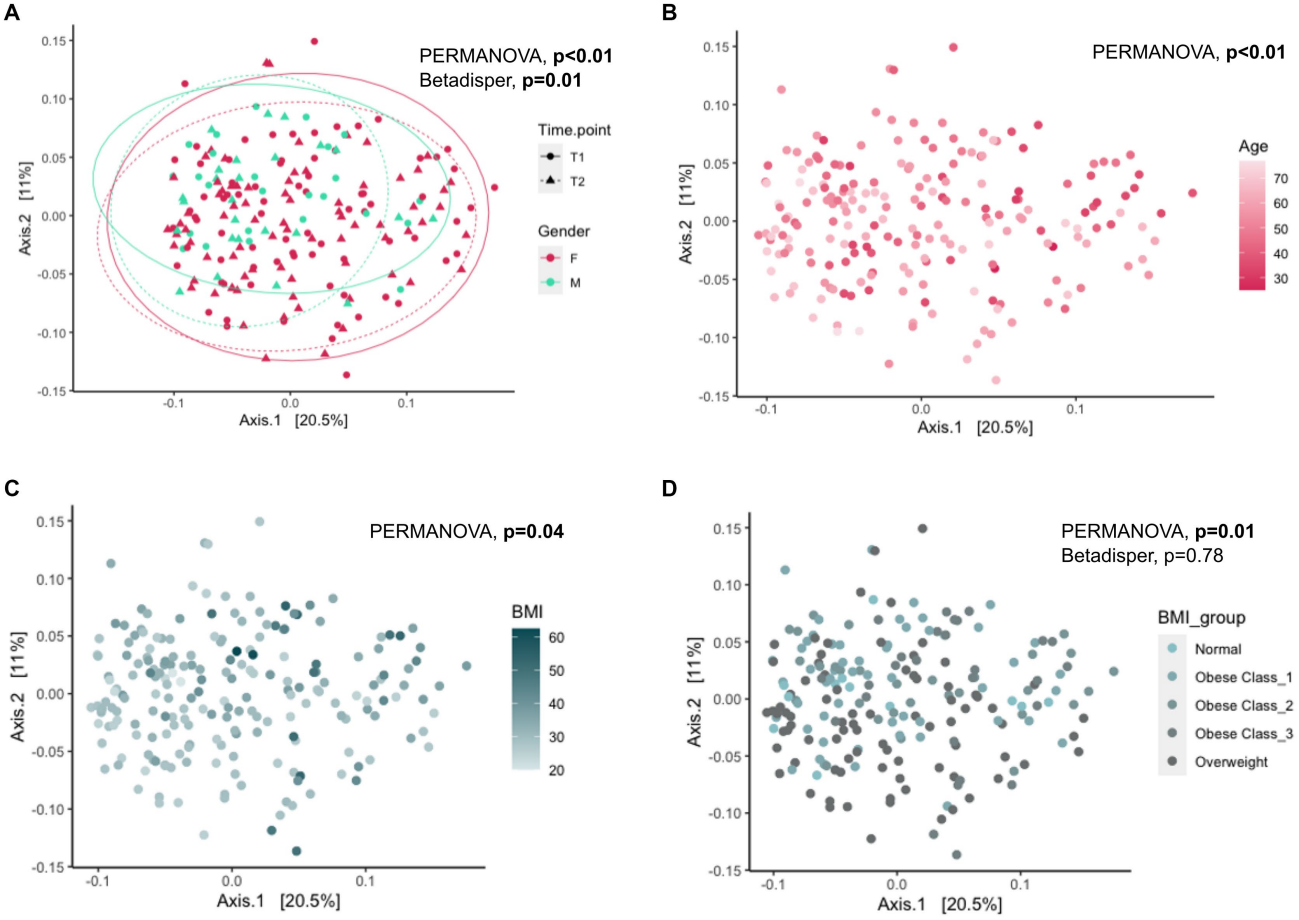
Association of Gender, Age, and BMI with gut microbial diversity. Principal Coordinate Analysis (PCoA) plot showing the beta diversity of the gut microbes across all individuals based on the Bray–Curtis dissimilarity. Ellipses in panel **(A)** represent 95% confidence regions stratified by gender at each time point. PERMANOVA analysis showed significant differences (shift) in overall diversity **(A)** between gender (PERMANOVA: *p*-value <0.01, Betadisper: Homogeneity of Variances test: *p*-value = 0.01), **(B)** with age (PERMANOVA: *p*-value <0.01), **(C)** with BMI (PERMANOVA: *p*-value = 0.04) and **(D)** between BMI groups (PERMANOVA, *p*-value = 0.01, Betadisper: Homogeneity of Variances test: *p*-value = 0.78). PERMANOVA: Permutational Multivariate Analysis of Variance; T1: Early phase; T2: Follow-up phase.

We used multivariate association methods to evaluate the relative contribution of different factors to the interindividual differences in gut microbiome profiles. We identified Age, BMI, Gender, Alcohol consumption, Antidepressants, or anxiolytics, and Time Point as significantly associated with the overall gut microbial diversity (*p*-values <0.05; see Table 2, Figures 1B,D, Figures 2B–D). Interestingly, the magnitude of the effect of BMI was 5.8 times larger than that of Time Point, suggesting that from the intervention point of view, the change in BMI is the main correlate of microbiome change. It is also noteworthy that the interaction between BMI and Time Point was not significant (*p*-value = 0.23), indicating that the overall correlation between the microbiome composition and BMI was not significantly different between the T1 and T2 samples. Interestingly, there was a significant difference in inter-individual variation between the genders (Male vs. Female, Betadipser test *p*-value = 0.01), which reduced at T2 (Figure 2A).

**Table 2:** Results from multivariate analysis (PERMANOVA) illustrate an association between variables and gut microbial diversity.

Description	Df	SumOfSqs	*R* ^2^	*F*	Pr(>F)
Gender	1	0.0692	0.01444	3.202	0.00194**
Age	1	0.1146	0.02389	5.2991	0.00194**
Time.point	1	0.0169	0.00352	0.7801	0.00026***
BMI	1	0.0984	0.02051	4.5505	0.04332*
comorbidity_binary	1	0.0331	0.00689	1.5285	0.18537
FGID_binary	1	0.0295	0.00616	1.3662	0.06771
Antidepressants_anxiolytics	1	0.0356	0.00743	1.6483	0.01376*
Alcohol consumption	1	0.0581	0.01212	2.6879	0.01449*
Recreational_drugs	1	0.0584	0.01217	2.6994	0.66618
Gender:BMI	1	0.0291	0.00608	1.348	0.89259
Age:BMI	1	0.0487	0.01016	2.254	0.48809
Time point:BMI	1	0.0071	0.00148	0.3281	0.23059
BMI:Alcohol consumption	1	0.039	0.00814	1.8058	0.81954
BMI:Recreational drugs	1	0.0285	0.00594	1.3185	0.70696

PERMANOVA: Permutational Multivariate Analysis of Variance.

We also tested the influence of these predictors on Shannon’s and Simpson’s diversity and identified a significant positive association between Age and Shannon’s (*p*-value = 1.6×10^−4^) and Simpson’s (*p*-value = 2.9×10^−2^) diversity and a negative association between BMI and Shannon’s (*p*-value = 2.7×10^−3^) but not with Simpson’s (*p*-value = 0.39) diversity (Supplementary Table S2).

### Gut microbial genera and pathways are associated with weight loss

In total, 36 genera showed a statistically significant association with BMI after correcting for multiple testing (FDR < 0.05; Supplementary Table S3). Of note, *Megasphaera, Acidaminococcus, Roseburia,* and members of the Lachnocpiraceae family (including *Lachnoclostridium, Sellimonas, Tyzzerella,* and unannotated genera Lachnospiraceae UCG-001) were associated with increasing BMI. While *Desulfovibrio, Solobacterium, Phascolarctobacterium*, Christensenellaceae R7 group, Alistipes, Clostridia vadinBB60 group, *Akkermansia,* and unannotated genera from Oscillospiraceae family (including UCG_003 and UCG_005,) were associated with lower BMI. We found that the abundance of 24 pathways was significantly associated with BMI (FDR < 0.05; Table 3; Supplementary Table S4). These pathways were functionally related, and several were associated with particular classes of metabolites, including the degradation of simple sugars (carbohydrates) such as arabinose (MF0014), sucrose (MF0010), and melibiose (MF0009), along with fructan (MF0002) and arabinoxylan, which were significantly associated with higher BMI. Biosynthesis of propionate (MGB054 and MGB055), a Short Chain Fatty Acid (SCFA), GABA (γ-Aminobutyric acid) synthesis (MGB021), putrescine degradation (MF0082), mucin degradation (MF0103), and degradation of amino acids such as lysine (MF0057) and serine (MF0048) were associated with lower BMI. We also identified microbial genera and pathways associated with significant changes between T1 vs. T2 which provides evidence of the intervention’s ability to modulate the gut microbiome composition (see Supplementary Tables S3, S4).

**Table 3:** Association of gut microbial pathways and BMI.

Module	Pathway name	Estimate	Std. Error	*t* value	*p*-value	FDR
Positive association with BMI						
MF0043	Cysteine biosynthesis/homocysteine degradation	0.0205	0.0038	5.4278	2.54E-07	1.61E-05
MF0102	Sulfate reduction (dissimilatory)	0.0106	0.0020	5.3322	4.01E-07	8.33E-06
MF0098	Hydrogen metabolism	0.0093	0.0024	3.9016	0.0001	0.0035
MF0001	Arabinoxylan degradation	0.0089	0.0017	5.1680	7.34E-07	1.51E-05
MF0041	Valine degradation I	0.0049	0.0013	3.8299	0.0002	0.0036
MF0078	Lactaldehyde degradation	0.0046	0.0009	5.2245	7.24E-07	9.12E-06
MF0055	Arginine degradation V	0.0046	0.0011	4.0972	6.94E-05	0.0014
MF0009	Melibiose degradation	0.0045	0.0010	4.6754	6.32E-06	0.0001
MF0014	Arabinose degradation	0.0040	0.0011	3.6626	0.0004	0.0069
MF0002	Fructan degradation	0.0036	0.0010	3.5593	0.0005	0.0103
MF0010	Sucrose degradation I	0.0027	0.0007	4.1785	4.97E-05	0.0013
Negative association with BMI						
MF0071	Pentose phosphate pathway (non-oxidative branch)	−0.0012	0.0004	−3.4249	0.0008	0.0360
MF0048	Serine degradation	−0.0030	0.0006	−5.3007	4.95E-07	7.70E-06
MF0096	Succinate production	−0.0061	0.0018	−3.3307	0.0011	0.0185
MGB054	Propionate synthesis II	−0.0073	0.0024	−3.0817	0.0025	0.0387
MF0080	Lactate consumption II	−0.0073	0.0024	−3.0817	0.0025	0.0387
MF0103	Mucin degradation	−0.0080	0.0020	−4.0253	9.73E-05	0.0020
MGB047	Acetate degradation	−0.0083	0.0028	−3.0072	0.0032	0.0493
MF0075	Acetate to acetyl-CoA	−0.0083	0.0028	−3.0072	0.0032	0.0493
MGB055	Propionate synthesis III	−0.0112	0.0030	−3.7771	2.32E-04	6.64E-03
MF0057	Lysine degradation I	−0.0131	0.0030	−4.4138	1.94E-05	0.0008
MGB038	Inositol degradation	−0.0138	0.0041	−3.3388	1.07E-03	0.0328
MGB021	GABA synthesis II	−0.0277	0.0056	−4.9627	2.26E-06	0.0003
MF0082	Putrescine degradation	−0.0277	0.0056	−4.9627	2.26E-06	0.0003

We analyzed the intersection between the genera associated with BMI and Time Point (T2 vs. T1) and identified 8 out of the 36 (22%) taxa with statistically significant changes in both analyses. Interestingly, these 8 taxa had a pattern of association indicating that the weight loss program improved their abundance, i.e., negatively associated with BMI and increased at T2 or positively associated with BMI and decreased at T2. In particular, those negatively associated with BMI, *Desulfovibrio*, *Solobacterium*, Christensenellaceae R7 group, *Anaerotruncus*, *Parabacteroides*, Oscillospiraceae UCG-002, were enriched at time point T2; on the other hand, genera positively associated with BMI*, Lachnoclostrdium* and *Roseburia* depleted substantially at T2 (See Supplementary Figure S2A; Supplementary Table S3). We also observed 7 genera with statistically significant association with BMI (FDR < 0.05) and marginal evidence of changing abundance at T2 (*p*-value <0.05), including *Akkermansia*, Clostridia vadinBB60 group, Oscillibacter, Oscillospiraceae UCG-005, *Phascolarctobacterium*, *Ruminococcus gauvreauii* group and Unannotated Oscillospiraceae (Family). All of these, except for Phascolarctobacterium, had a directional change at T2 indicating the intervention improved their abundance (Supplementary Table S3).

Similarly, four pathways were significantly associated with BMI and Time Point, including MF0043 (cysteine biosynthesis/homocysteine degradation), MF0078 (lactaldehyde degradation), MF0096 (succinate production), and MGB038 (Inositol degradation), and another six reached marginal evidence of changing abundance at T2 (*p*-values <0.05), including melibiose degradation (MF0009), arginine degradation V (MF0055), lysine degradation I (MF0057), acetate to acetyl-CoA (MF0075), mucin degradation (MF0103), and acetate degradation (MGB047). We found that all of these pathways changed at T2, indicating that the weight loss program had improved their abundance (Supplementary Table S4).

### Association of gut microbiome networks with BMI

**Figure 3:**
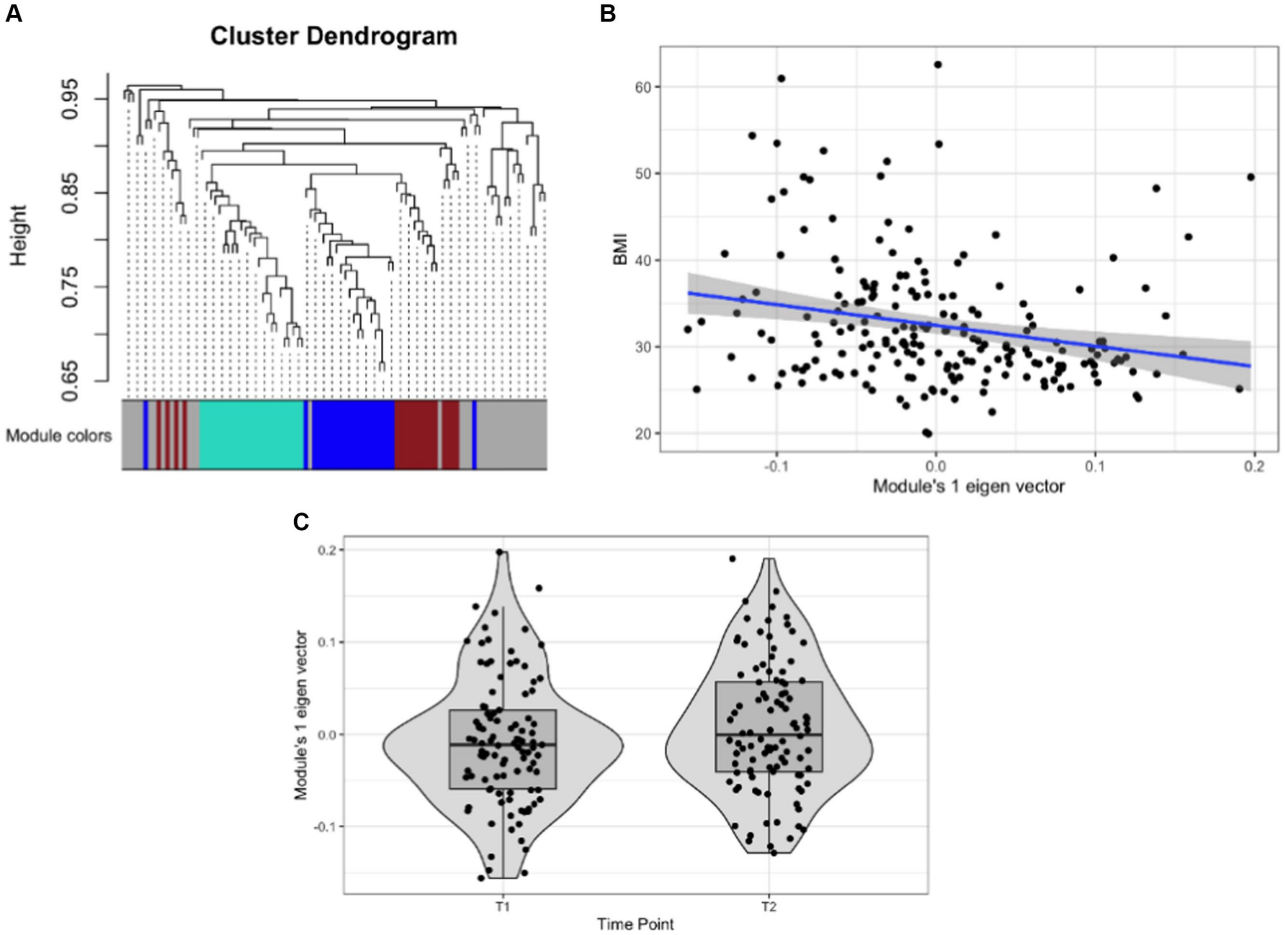
Association of gut microbial network modules and BMI. **(A)** The dendrogram presents the correlation among gut microbiome taxa abundance values on which each tip of the tree represents a genus, and two genera are close to each other if they have a high signed bicor correlation value. Color blocks at the lower part of the figure represent the groups of taxa network modules identified as independent network modules. The relationship between colors and numbers is the following: turquoise = 1, blue = 2, brown = 3, and gray = 0. **(B)** Correlation between module’s 1 eigenvector and BMI. The regression line (blue line and the gray area covers 1 standard deviation around the mean) represents the linear relationship between the two variables. **(C)** box-plot displaying the module’s 1 eigenvector values across T1 and T2.

Weighted network analyses identified three network modules as the study samples’ overall organizational structure of the gut microbiome (See Figure 3A; Table 4). These network modules ranged in size between 18 to 24 taxa. We used the module’s eigenvector as a summary of the abundance of the taxa of each module and tested its statistical association with BMI and the change between T1 to T2. Module 1 was associated with BMI with a negative correlation (Sidak *p*-value = 0.013; Figure 3B). Module 1 also showed a change in the abundance pattern between T1 and T2 with a positive correlation (Sidak p-value = 0.045; Figure 3C). Several genera identified in univariate analyses were part of and key drivers of Module 1 variation such as the unannotated genus of Oscillospiraceae (Family) including Oscillospiraceae UCG-002 and UCG-005, unannotated genus of Lachnospiraceae (Family) along with genera of unannotated genus of Ruminococcaceae (Family), Anaerovoracaceae Family XIII AD3011 group, Clostridia Vadin BB60 group, Christensenellaceae R-7 group, Clostridia vadinBB60 group, *Alistipes*, and *Roseburia*. Supplementary Table S5 lists the taxa associated with network modules 1 and 2 and their correlation with their module’s eigenvector. Supplementary Figure S1 presents the correlation pattern between the Module 1 eigenvector and its top three driving genera.

**Table 4:** Association of gut microbial network modules with BMI and time point.

Module name	Module color	Estimate	Std. Error	df	*t* value	*p*-value	Sidak *p*-value
BMI							
ME1	Turquoise	−0.0023	0.0008	−2.91	116.00	0.0043	0.0130
ME2	Blue	−0.0004	0.0003	−1.38	128.00	0.1690	0.4261
ME3	Brown	0.0006	0.0009	0.68	127.00	0.5000	0.8750
Time point							
ME1	Turquoise	0.0163	0.0066	2.47	99.80	0.0153	0.0452
ME2	Blue	0.0044	0.0022	2.00	99.40	0.0479	0.1369
ME3	Brown	−0.0048	0.0061	−0.79	100.00	0.4320	0.8167

## Discussion

Our study analyzed a retrospective cohort of 103 individuals from their enrollment (T0) to study the effect of Digbi Health’s digital therapeutic obesity management and weight loss program on their BMI and gut microbiome (measured at T1 and T2) as primary endpoints. Our study participants experienced significant weight loss, with a mean decrease of 2.6 BMI units from T0 to T2 and 1.4 BMI units from T1 to T2 (Table 1). This weight loss has notable health and economic implications, particularly given that a substantial proportion of individuals lost significant percentages of their body weight (36). The individual’s demographics or lifestyle factors analyzed did not influence the weight loss (or gain) achieved between T1 and T2 (Supplementary Table S1). This is in line with our previous scientific reports that show equitable results of Digbi Health’s weight loss program concerning the individual gender and age, among other socio-economic factors (28). Overall, we observed a significant improvement in the gut microbiome diversity between T1 and T2 and this was primarily associated with weight loss (Table 2; Supplementary Table S2). Additionally, we identified gut microbiome genera (Supplementary Table S3), bacterial functional pathways (Supplementary Table S4), and bacterial community networks (Table 4; Supplementary Table S5) associated with weight loss.

Consistent with previous studies, our findings show that decreasing BMI is associated with increased gut microbial diversity (37–40). Notably, the change in beta diversity is significantly more influenced by BMI than other factors such as medication intake or alcohol consumption, underscoring the pivotal role of BMI in shaping gut microbiome diversity (Table 2; Supplementary Table S2).

Our analysis identified significant associations between BMI and the abundance of certain genera and microbial pathways. Notably, the intervention appears to have beneficially modulated the abundance of genera like Christensenella and Oscillospiraceae, which have known associations with BMI (41). The observed increase in mucin degradation pathways, linked to Akkermansia, aligns with previous studies highlighting its role in metabolic health improvement (42).

The association of simple sugar metabolism pathways with higher BMI supports the notion that certain gut microbiome compositions can enhance energy extraction from food, contributing to obesity (17, 43). This finding is consistent with previous studies suggesting that the microbiome’s efficiency in extracting energy from simple sugars plays a role in weight gain (17, 43, 44).

Conversely, propionate synthesis pathways, including MGB055 and MGB054, showed a negative association with BMI (Table 3). Propionate is a type of SCFA produced by certain types of bacteria in the gut. Increased gut propionate levels have been linked to reduced inflammation, improved insulin sensitivity, and regulation of appetite and body weight maintenance by promoting the secretion of Peptide YY (PYY) and Glucagon-like peptide-1 (GLP-1) (45, 46). Evidence of more than a decade of research has shown that gut microbiome alterations (both at taxa and functional levels) are associated with diet-induced obesity and are reversible, leading to the suggestion that weight loss programs targeting the gut microbiome can be used to treat obesity (3, 8, 47). In this context, the evidence presented in this and our previous studies supports this premise. It highlights microbial taxa and their functions that may be targeted to improve the gut microbiome composition with concomitant positive effects on health outcomes, including obesity, cardiovascular health, mental health, and functional gastrointestinal disorders (26–29).

Interestingly, the 8 genera associated with BMI, also changed their abundance in a pattern consistent with the beneficial effect of the weight loss program on the gut microbiome. In particular, their abundance was negatively associated with BMI, which increased at T2, or their abundance was positively associated with BMI and decreased at T2. Further, four pathways, including lactaldehyde degradation (MF0078), Inositol degradation (MGB038), succinate production (MF0096), and cysteine biosynthesis/homocysteine degradation (MF0043) were seen to have a similar trend (Supplementary Table S4). These pathways are associated with insulin signaling and lipid metabolism, which play critical roles in overall metabolic health (48).

The gut microbiome is a dynamic assemblage of ecological communities whose components are physically and metabolically interacting. We carried out network analyses to identify groups of taxa with tightly correlated abundance patterns that may reflect the underlying microbial community assembly and are associated with human health outcomes (49, 50). Our analyses identified three network modules of which Module 1 was significantly associated with BMI with a negative correlation (Figure 3B; Supplementary Table S5). Reassuringly, several of the genera in this module associated with BMI and Time Point with the same directions of effect, have been associated with BMI in previous studies, including, *Roseburia*, *Butyricicoccus*, and *Lachnospira*, Christensenellaceae, *Alistipes,* and *Sutterella* (51–53) (Supplementary Table S3).

These findings provide further evidence that the composition of the gut microbiome is associated with BMI (or obesity) and that the changes in BMI are reflected in changes in abundance in microbial communities (networks), genera, and their functional pathways. We identified several gut microbial components (genera, functional pathways, and communities) that significantly changed during the intervention period evaluated (T1 to T2) and whose changes are in concordance with an intervention-induced weight loss. This provides evidence of the longitudinal impact the intervention has on the gut microbiome. The gut microbiome markers identified, warrant further research and may prove useful as biomarkers to assess the response of the gut microbiome to dietary interventions, the effect of medications, or individual food components aimed at supporting weight loss.

This study has some limitations that are important to note. The findings presented here are derived from a weight loss cohort and thus may not reflect the relationship between BMI and gut microbiome diversity and health in the broader population. This was a retrospective observational study that did not collect information regarding longitudinal changes in factors that influence the microbiome composition, such as disease diagnoses, medication changes, stress levels, or measures of environmental, social, and work determinants of health which may also explain the gut microbiome changes observed. Our study did not include a control group, because all enrolled individuals underwent the weight loss intervention, and we cannot rule out that the association reported may change in magnitude or direction in individuals not undergoing a weight loss program or a different dietary intervention. This study employed a two-time point design, limiting our ability to capture the dynamic fluctuations of the gut microbiome throughout the intervention. Capturing data at multiple time points could provide a more nuanced understanding of how the microbiome adapts and responds to dietary changes. However, it still offers a snapshot of the association between dietary changes, gut microbiome and weight loss from a real-world dietary intervention. Our associations with bacterial functional pathways relied on predicting the abundance of the relevant genes and did not directly relate to the activity at the enzymatic or molecular level. There were substantial variations in the time elapsed between T0 to T2, and T1 to T2. Despite not finding a relationship between these periods and the amount of weight loss, we cannot rule out a systematic effect on the changes in the microbiome observed. These limitations imply that our results may be affected by ascertainment or collider biases that we cannot effectively control due to this study’s observational and retrospective nature. Nonetheless, our findings provide new insights into the effects of dietary and lifestyle changes on the longitudinal evolution of the gut microbiome and its potential involvement in weight loss and warrant consideration by the broader scientific community.

In conclusion, this study reinforces the concept of a dynamic link between the gut microbiome and body weight. We observed significant weight loss accompanied by an improvement in gut microbiome diversity following a real-world, 6-month dietary intervention. Furthermore, our results suggest specific gut microbial communities, genera, and functional pathways are associated with weight loss, highlighting the potential for microbiome-targeted strategies in obesity treatment. Future research with a prospective design and broader participant demographics can strengthen these associations and elucidate causal relationships between gut microbiota, dietary interventions, and weight management. Nonetheless, this study provides valuable insights from a real-world setting, offering a snapshot of how dietary changes can influence gut microbiome composition and contribute to weight loss. These findings pave the way for further exploration of personalized dietary interventions that manipulate the gut microbiome for weight management and overall health promotion.

## Data availability statement

The datasets presented in this study can be found in online repositories. The microbiome sequence data used in this study were submitted to NCBI SRA under Bioproject accession number PRJNA907500. The names of the repository/repositories and accession number(s) can be found in the article/Supplementary material.

## Ethics statement

The studies involving humans were approved by the Institutional Review Board of E&I Review Services (protocol code #18053 on 05/22/2018). The study was conducted in accordance with the Declaration of Helsinki. The studies were conducted in accordance with the local legislation and institutional requirements. The participants provided their written informed consent to participate in this study.

## Author contributions

SK: Data curation, Formal analysis, Investigation, Methodology, Resources, Software, Validation, Visualization, Writing – original draft, Writing – review & editing. IP: Data curation, Formal analysis, Investigation, Methodology, Resources, Software, Validation, Visualization, Writing – original draft, Writing – review & editing. BJ: Data curation, Formal analysis, Software, Writing – review & editing. KM: Data curation, Formal analysis, Software, Writing – review & editing. SS: Formal analysis, Methodology, Writing – review & editing. CI: Formal analysis, Methodology, Writing – review & editing. GK: Conceptualization, Writing – review & editing. PD: Conceptualization, Writing – review & editing. RS: Conceptualization, Funding acquisition, Writing – review & editing. DA: Conceptualization, Project administration, Supervision, Writing – original draft, Writing – review & editing.

## References

[1] Keller M Svensson SIA Rohde-Zimmermann K Kovacs P Böttcher Y. Genetics and epigenetics in obesity: what do we know so far? Curr Obes Rep. (2023) 12:482–501. doi: 10.1007/s13679-023-00526-z, PMID: 37819541 PMC10748780

[2] Albuquerque D Nóbrega C Manco L Padez C. The contribution of genetics and environment to obesity. Br Med Bull. (2017) 123:159–73. doi: 10.1093/bmb/ldx022 28910990

[3] Turnbaugh PJ Bäckhed F Fulton L Gordon JI. Diet-induced obesity is linked to marked but reversible alterations in the mouse distal gut microbiome. Cell Host Microbe. (2008) 3:213–23. doi: 10.1016/j.chom.2008.02.015, PMID: 18407065 PMC3687783

[4] Louis S Tappu R-M Damms-Machado A Huson DH Bischoff SC. Characterization of the gut microbial Community of Obese Patients Following a weight-loss intervention using whole metagenome shotgun sequencing. PLoS One. (2016) 11:e0149564. doi: 10.1371/journal.pone.0149564, PMID: 26919743 PMC4769288

[5] Kahleova H Rembert E Alwarith J Yonas WN Tura A Holubkov R. Effects of a low-fat vegan diet on gut microbiota in overweight individuals and relationships with body weight, body composition, and insulin sensitivity. A randomized clinical trial. Nutrients. (2020) 12:2917. doi: 10.3390/nu12102917 32987642 PMC7598634

[6] Diener C Qin S Zhou Y Patwardhan S Tang L Lovejoy JC. Baseline gut metagenomic functional gene signature associated with variable weight loss responses following a healthy lifestyle intervention in humans. mSystems. (2021) 6:e0096421. doi: 10.1128/mSystems.00964-21, PMID: 34519531 PMC8547453

[7] Cho I Blaser MJ. The human microbiome: at the interface of health and disease. Nat Rev Genet. (2012) 13:260–70. doi: 10.1038/nrg3182 22411464 PMC3418802

[8] David LA Maurice CF Carmody RN Gootenberg DB Button JE Wolfe BE. Diet rapidly and reproducibly alters the human gut microbiome. Nature. (2014) 505:559–63. doi: 10.1038/nature12820, PMID: 24336217 PMC3957428

[9] David LA Materna AC Friedman J Campos-Baptista MI Blackburn MC Perrotta A. Host lifestyle affects human microbiota on daily timescales. Genome Biol. (2014) 15:R89. doi: 10.1186/gb-2014-15-7-r89, PMID: 25146375 PMC4405912

[10] Bik EM Ugalde JA Cousins J Goddard AD Richman J Apte ZS. Microbial biotransformations in the human distal gut: microbial biotransformations in the distal gut. Br J Pharmacol. (2018) 175:4404–14. doi: 10.1111/bph.14085, PMID: 29116650 PMC6255956

[11] Heianza Y Sun D Li X DiDonato JA Bray GA Sacks FM. Gut microbiota metabolites, amino acid metabolites and improvements in insulin sensitivity and glucose metabolism: the POUNDS lost trial. Gut. (2019) 68:263–70. doi: 10.1136/gutjnl-2018-316155, PMID: 29860242 PMC6275143

[12] Ding S Xue J Zhang Q Zheng L. Trimethylamine-N-oxide is an important target for heart and brain diseases. Med Rev. (2022) 2:321–3. doi: 10.1515/mr-2022-0026, PMID: 37724327 PMC10388736

[13] Fan Y Pedersen O. Gut microbiota in human metabolic health and disease. Nat Rev Microbiol. (2021) 19:55–71. doi: 10.1038/s41579-020-0433-9 32887946

[14] Bailén M Bressa C Martínez-López S González-Soltero R Montalvo Lominchar MG San Juan C. Microbiota features associated with a high-fat/low-Fiber diet in healthy adults. Front Nutr. (2020) 7:583608. doi: 10.3389/fnut.2020.583608, PMID: 33392236 PMC7775391

[15] Wang B Kong Q Li X Zhao J Zhang H Chen W. A high-fat diet increases gut microbiota biodiversity and energy expenditure due to nutrient difference. Nutrients. (2020) 12:3197. doi: 10.3390/nu12103197 33092019 PMC7589760

[16] Grembi JA Nguyen LH Haggerty TD Gardner CD Holmes SP Parsonnet J. Gut microbiota plasticity is correlated with sustained weight loss on a low-carb or low-fat dietary intervention. Sci Rep. (2020) 10:1405. doi: 10.1038/s41598-020-58000-y, PMID: 31996717 PMC6989501

[17] Turnbaugh PJ Ley RE Mahowald MA Magrini V Mardis ER Gordon JI. An obesity-associated gut microbiome with increased capacity for energy harvest. Nature. (2006) 444:1027–31. doi: 10.1038/nature05414, PMID: 17183312

[18] Le Chatelier E Nielsen T Qin J Prifti E Hildebrand F Falony G. Richness of human gut microbiome correlates with metabolic markers. Nature. (2013) 500:541–6. doi: 10.1038/nature12506 23985870

[19] Kootte RS Vrieze A Holleman F Dallinga-Thie GM Zoetendal EG de Vos WM. The therapeutic potential of manipulating gut microbiota in obesity and type 2 diabetes mellitus. Diabetes Obes Metab. (2012) 14:112–20. doi: 10.1111/j.1463-1326.2011.01483.x, PMID: 21812894

[20] Zeevi D Korem T Zmora N Israeli D Rothschild D Weinberger A. Personalized nutrition by prediction of glycemic responses. Cell. (2015) 163:1079–94. doi: 10.1016/j.cell.2015.11.001 26590418

[21] Balfegó M Canivell S Hanzu FA Sala-Vila A Martínez-Medina M Murillo S. Effects of sardine-enriched diet on metabolic control, inflammation and gut microbiota in drug-naïve patients with type 2 diabetes: a pilot randomized trial. Lipids Health Dis. (2016) 15:78. doi: 10.1186/s12944-016-0245-0, PMID: 27090218 PMC4836051

[22] Jian C Luukkonen P Sadevirta S Yki-Jarvinen H Salonen A. Impact of short-term overfeeding of saturated or unsaturated fat or sugars on the gut microbiota in relation to liver fat in obese and overweight adults. Clin Nutr. (2021) 40:207–16. doi: 10.1016/j.clnu.2020.05.008 32536582

[23] Chen M Sun Q Giovannucci E Mozaffarian D Manson JE Willett WC. Dairy consumption and risk of type 2 diabetes: 3 cohorts of US adults and an updated meta-analysis. BMC Med. (2014) 12:215. doi: 10.1186/s12916-014-0215-1, PMID: 25420418 PMC4243376

[24] Ohlsson B. An Okinawan-based Nordic diet improves glucose and lipid metabolism in health and type 2 diabetes, in alignment with changes in the endocrine profile, whereas zonulin levels are elevated (review). Exp Ther Med. (2019) 17:2883–93. doi: 10.3892/etm.2019.7303 30936958 PMC6434283

[25] Frost F Storck LJ Kacprowski T Gärtner S Rühlemann M Bang C. A structured weight loss program increases gut microbiota phylogenetic diversity and reduces levels of Collinsella in obese type 2 diabetics: a pilot study. PLoS One. (2019) 14:e0219489. doi: 10.1371/journal.pone.0219489, PMID: 31318902 PMC6638920

[26] Sinha R Kachru D Ricchetti RR Singh-Rambiritch S Muthukumar KM Singaravel V. Leveraging genomic associations in precision digital Care for Weight Loss: cohort study. J Med Internet Res. (2021) 23:e25401. doi: 10.2196/25401, PMID: 33849843 PMC8173391

[27] Ricchetti RR Sinha R Muthukumar KM Singh-Rambiritch S Underwood B Junaid I. Outcomes of a precision digital care program for obesity and associated comorbidities: results in real world clinical practice. Int J Clin Med Cases. (2020) 3:11. doi: 10.31021/ijcmc.20203160

[28] Pedroso I Kumbhare SV Joshi B Saravanan SK Mongad DS Singh-Rambiritch S. Mental health symptom reduction using digital therapeutics care informed by genomic SNPs and gut microbiome signatures. J Pers Med. (2022) 12:1237. doi: 10.3390/jpm12081237, PMID: 36013186 PMC9409755

[29] Kumbhare SV Francis-Lyon PA Kachru D Uday T Irudayanathan C Muthukumar KM. Digital therapeutics care utilizing genetic and gut microbiome signals for the Management of Functional Gastrointestinal Disorders: results from a preliminary retrospective study. Front Microbiol. (2022) 13:826916. doi: 10.3389/fmicb.2022.826916, PMID: 35391720 PMC8983270

[30] Bolyen E Rideout JR Dillon MR Bokulich NA Abnet CC Al-Ghalith GA. Reproducible, interactive, scalable and extensible microbiome data science using QIIME 2. Nat Biotechnol. (2019) 37:852–7. doi: 10.1038/s41587-019-0209-9, PMID: 31341288 PMC7015180

[31] Xia Y. *q2-repeat-rarefy: QIIME2 plugin for generating the average rarefied table for library size normalization using repeated rarefaction*. (2021). Available at: https://github.com/yxia0125/q2-repeat-rarefy

[32] Valles-Colomer M Falony G Darzi Y Tigchelaar EF Wang J Tito RY. The neuroactive potential of the human gut microbiota in quality of life and depression. Nat Microbiol. (2019) 4:623–32. doi: 10.1038/s41564-018-0337-x, PMID: 30718848

[33] Douglas GM Maffei VJ Zaneveld JR Yurgel SN Brown JR Taylor CM. PICRUSt2 for prediction of metagenome functions. Nat Biotechnol. (2020) 38:685–8. doi: 10.1038/s41587-020-0548-6 32483366 PMC7365738

[34] Darzi Y Falony G Vieira-Silva S Raes J. Towards biome-specific analysis of meta-omics data. ISME J. (2016) 10:1025–8. doi: 10.1038/ismej.2015.188, PMID: 26623543 PMC5029225

[35] Aitchison J. The statistical analysis of compositional data. New York, USA: Chapman and Hall (1986). 416 p.

[36] Ding Y Fan X Blanchette CM Smolarz BG Weng W Ramasamy A. Economic value of nonsurgical weight loss in adults with obesity. J Manag Care Spec Pharm. (2021) 27:37–50. doi: 10.18553/jmcp.2020.20036, PMID: 33164723 PMC10394211

[37] Lv Y Qin X Jia H Chen S Sun W Wang X. The association between gut microbiota composition and BMI in Chinese male college students, as analysed by next-generation sequencing. Br J Nutr. (2019) 122:986–95. doi: 10.1017/S0007114519001909, PMID: 31397240

[38] Scheithauer TPM Rampanelli E Nieuwdorp M Vallance BA Verchere CB van Raalte DH. Gut microbiota as a trigger for metabolic inflammation in obesity and type 2 diabetes. Front Immunol. (2020) 11:571731. doi: 10.3389/fimmu.2020.571731, PMID: 33178196 PMC7596417

[39] Zouiouich S Loftfield E Huybrechts I Viallon V Louca P Vogtmann E. Markers of metabolic health and gut microbiome diversity: findings from two population-based cohort studies. Diabetologia. (2021) 64:1749–59. doi: 10.1007/s00125-021-05464-w, PMID: 34110438 PMC8245388

[40] Geng J Ni Q Sun W Li L Feng X. The links between gut microbiota and obesity and obesity related diseases. Biomed Pharmacother. (2022) 147:112678. doi: 10.1016/j.biopha.2022.112678 35134709

[41] Konikoff T Gophna U. Oscillospira: a central, enigmatic component of the human gut microbiota. Trends Microbiol. (2016) 24:523–4. doi: 10.1016/j.tim.2016.02.015, PMID: 26996766

[42] Depommier C Everard A Druart C Plovier H Van Hul M Vieira-Silva S. Supplementation with *Akkermansia muciniphila* in overweight and obese human volunteers: a proof-of-concept exploratory study. Nat Med. (2019) 25:1096–103. doi: 10.1038/s41591-019-0495-2, PMID: 31263284 PMC6699990

[43] Tilg H Moschen AR Kaser A. Obesity and the microbiota. Gastroenterology. (2009) 136:1476–83. doi: 10.1053/j.gastro.2009.03.030 19327360

[44] Turnbaugh PJ Hamady M Yatsunenko T Cantarel BL Duncan A Ley RE. A core gut microbiome in obese and lean twins. Nature. (2009) 457:480–4. doi: 10.1038/nature07540, PMID: 19043404 PMC2677729

[45] Tedelind S Westberg F Kjerrulf M Vidal A. Anti-inflammatory properties of the short-chain fatty acids acetate and propionate: a study with relevance to inflammatory bowel disease. World J Gastroenterol. (2007) 13:2826–32. doi: 10.3748/wjg.v13.i20.2826, PMID: 17569118 PMC4395634

[46] Chambers ES Viardot A Psichas A Morrison DJ Murphy KG Zac-Varghese SEK. Effects of targeted delivery of propionate to the human colon on appetite regulation, body weight maintenance and adiposity in overweight adults. Gut. (2015) 64:1744–54. doi: 10.1136/gutjnl-2014-307913, PMID: 25500202 PMC4680171

[47] Ley RE Turnbaugh PJ Klein S Gordon JI. Human gut microbes associated with obesity. Nature. (2006) 444:1022–3. doi: 10.1038/4441022a 17183309

[48] Atzeni A Bastiaanssen TFS Cryan JF Tinahones FJ Vioque J Corella D. Taxonomic and functional fecal microbiota signatures associated with insulin resistance in non-diabetic subjects with overweight/obesity within the frame of the PREDIMED-plus study. Front Endocrinol. (2022) 13:804455. doi: 10.3389/fendo.2022.804455, PMID: 35574036 PMC9097279

[49] Faust K Raes J. Microbial interactions: from networks to models. Nat Rev Microbiol. (2012) 10:538–50. doi: 10.1038/nrmicro2832 22796884

[50] Park CH Lee JG Lee A Eun CS Han DS. Network construction of gastric microbiome and organization of microbial modules associated with gastric carcinogenesis. Sci Rep. (2019) 9:12444. doi: 10.1038/s41598-019-48925-4, PMID: 31455798 PMC6712011

[51] Xu Z Jiang W Huang W Lin Y Chan FKL Ng SC. Gut microbiota in patients with obesity and metabolic disorders — a systematic review. Genes Nutr. (2022) 17:2. doi: 10.1186/s12263-021-00703-6 35093025 PMC8903526

[52] Zeng Q Li D He Y Li Y Yang Z Zhao X. Discrepant gut microbiota markers for the classification of obesity-related metabolic abnormalities. Sci Rep. (2019) 9:13424. doi: 10.1038/s41598-019-49462-w, PMID: 31530820 PMC6748942

[53] Parker BJ Wearsch PA Veloo ACM Rodriguez-Palacios A. The genus Alistipes: gut Bacteria with emerging implications to inflammation, Cancer, and mental health. Front Immunol. (2020) 11:906. doi: 10.3389/fimmu.2020.00906, PMID: 32582143 PMC7296073

