# Supplementary figures and images for: Longitudinal gut microbial signals are associated with weight loss: insights from a digital therapeutics program
Source: Front Nutr. 2024 Jul 8;11:1363079. doi: 10.3389/fnut.2024.1363079 (PMC11262244; doi:10.3389/fnut.2024.1363079)

A.

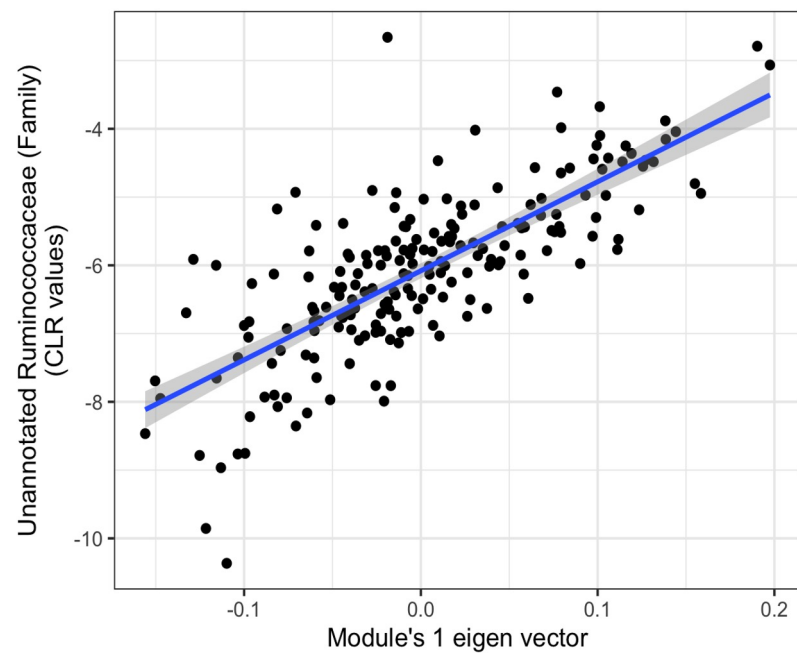

B.

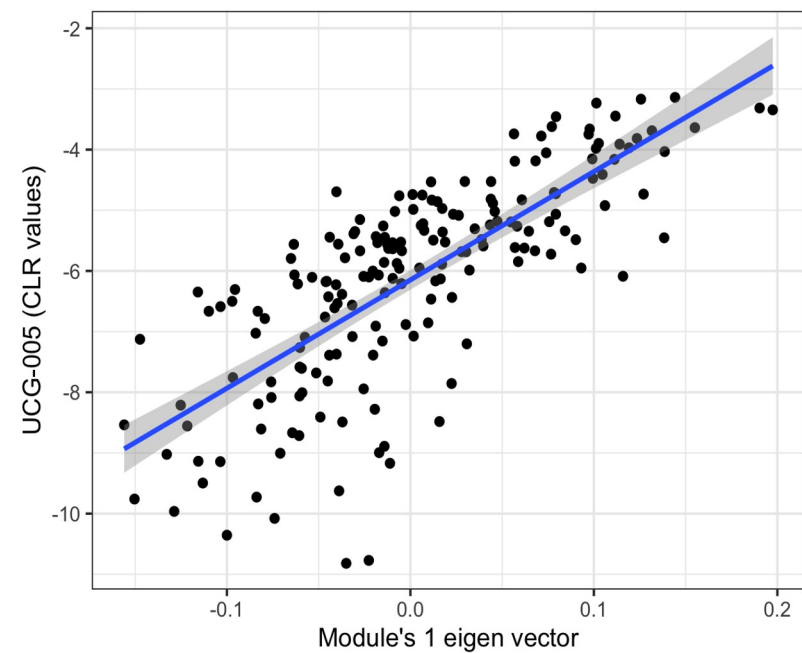

C.

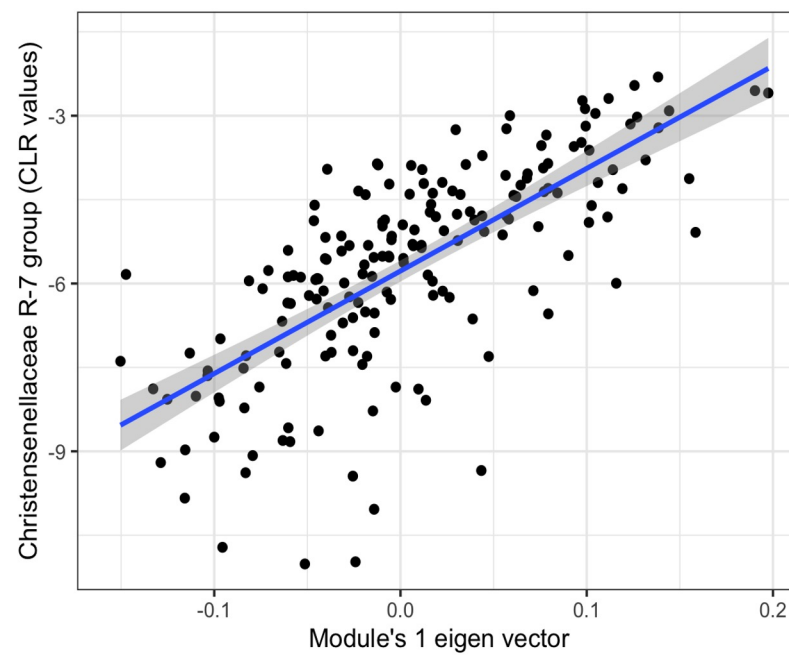

Supplement: Supplementary file 1 [file Image_1.PDF]

A.

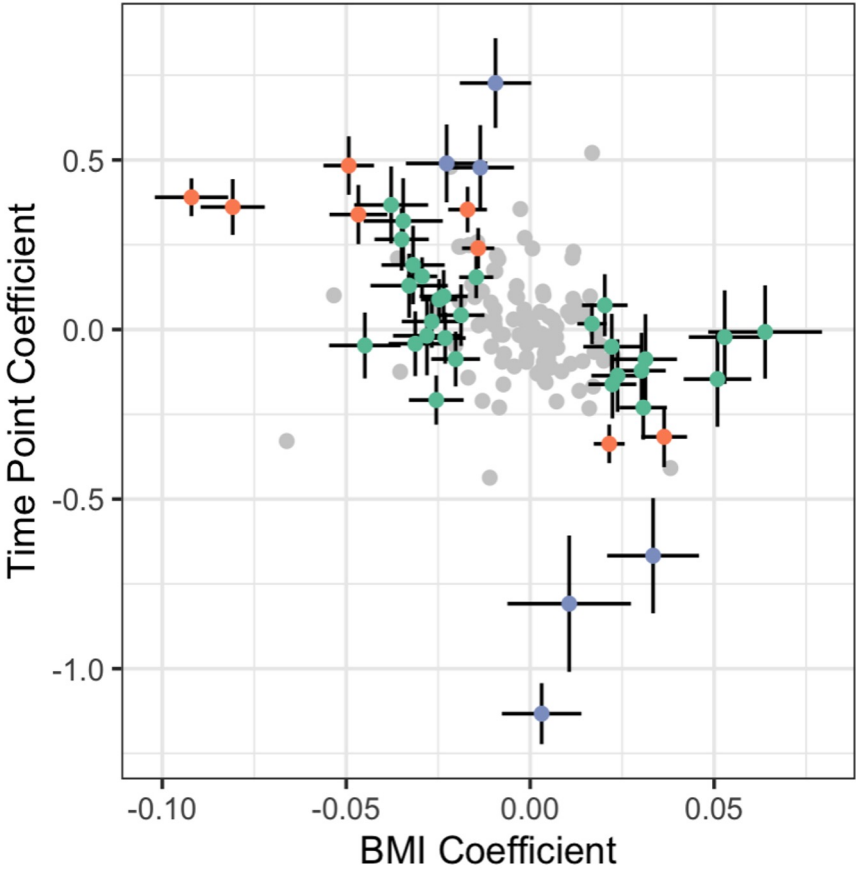

B.

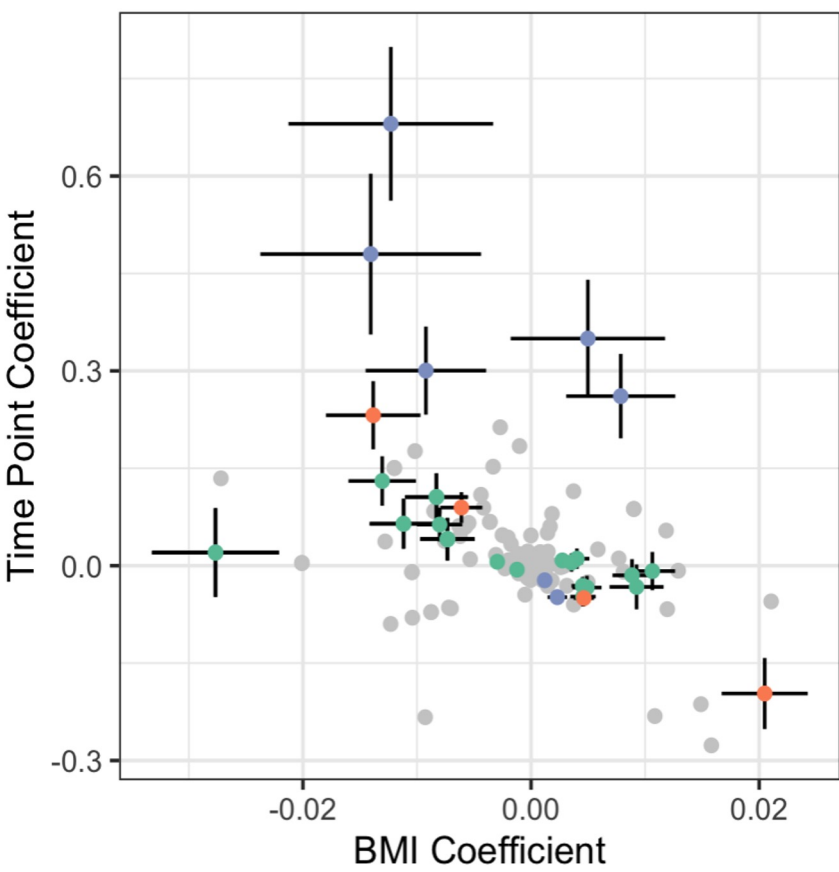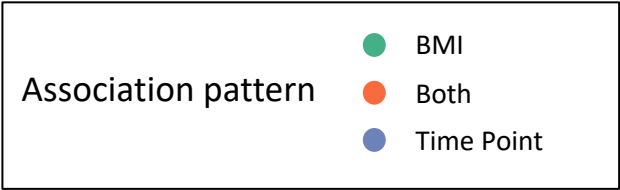

Supplement: Supplementary file 2 [file Image_2.PDF]
