# Supplementary material for: Longitudinal gut microbial signals are associated with weight loss: insights from a digital therapeutics program
Source: Front Nutr. 2024 Jul 8;11:1363079. doi: 10.3389/fnut.2024.1363079 (PMC11262244; doi:10.3389/fnut.2024.1363079)
Supplement: Supplementary file 3 [file Presentation_1.PDF]

# Longitudinal gut microbial signals are associated with weight loss: insights from a digital therapeutics program

Shreyas V. Kumbhare[1†], Inti Pedroso[1†], Bharat Joshi[1], Karthik M. Muthukumar[1], Santosh K. Saravanan[1], Carmel Irudayanathan[1], Gursimran S. Kochhar[2], Parambir S. Dulai[3], Ranjan Sinha[1], Daniel E. Almonacid[1*]

[1]Digbi Health, Mountain View, California, USA
[2]Division of Gastroenterology, Hepatology and Nutrition, Allegheny Health Network, Pittsburgh, Pennsylvania, USA
[3]Division of Gastroenterology, Northwestern University, Chicago, Illinois, USA
*Author to whom correspondence should be addressed: almonacid@digbihealth.com
†These authors contributed equally to this work

## Supplementary methods

### Network module analysis

We performed network analyses of the gut microbiome profiles to identify 1) network modules associated with BMI and BMI changes between the time points studied and 2) the key driving taxa of these network modules. We define network modules as a set of taxa with higher levels of correlation on their abundance among them compared to other taxa. We utilized the WGCNA software package for R (1). We preprocessed the taxa abundance table (unrarified sequencing counts) by calculating the CLR transformation and setting taxa with 0 counts as NA. We used the "pickSoftThreshold " function to explore the relationship between the power parameter and the connectivity and selected power = 1 for the analyses. Higher values led to a drastic reduction of average connectivity, which strongly influenced the detection of modules which translated into detecting none or one with values of the power parameter >3. For module detection we used the "blockwiseModules" function with the following non-default parameters: power=1, corType='bicor', corOptions=list(use='pairwise.complete.obs', checkMissingData=TRUE, TOMType="signed", minModuleSize= 10, reassignThreshold = 0, mergeCutHeight = 0.25, numericLabels=TRUE, pamRespectsDendro=FALSE, and replaceMissingAdjacencies=TRUE). The per taxa missingness values filtered out 52 taxa with an excess of missing values and for which pairwise correlations with other taxa could not be calculated. Likewise, 1 sample was removed from the analysis due to 50% missing values. We used the "bicor" correlation due to the non-normal distribution of microbiome abundance values. We compared the results obtained using the "bicor" correlation with a signed or unsigned TOMType and found that the resulting modules were identical. The

output of the “blockwiseModules" function provides an assignment of each taxon to a network module, a summary of the abundance patterns of the modules, and calculates the first eigenvector, which summarizes the main trend of the abundance matrix of the taxa associated with each module. We calculated the correlation between each taxon CLR abundance value and the module eigenvector to identify the taxa that are key drivers of the network module as described by the software authors (2).

### Statistical analysis

The main objective of Digbi Health’s lifestyle and dietary intervention is to achieve successful weight loss. As such, our primary focus of analyses was the correlations between weight loss (as measured by BMI) and gut microbiome composition at the genus level, microbial pathways, network modules, and microbial diversity indices. The longitudinal nature of this cohort also provided information regarding microbiome changes between T1 and T2 gut fecal samples, and these were informative of changes beyond weight, e.g., dietary changes or lifestyle changes. Therefore, we used the gut microbiome association with T1 vs T2 as a complement and to prioritize some of the associations found with BMI.

We used PERMANOVA to perform a multivariate association of individual’s covariates to inter-individual’s gut microbiome distance/similarity, measured using the Bray-Curtis dissimilarity, using the vegan package (adonis2 function, strata= user.id) in R. We also tested for homogeneity of multivariate dispersions (comparing inter-individual variations) between groups, we used betadisper test. We compared diversity indices between time points (T1 vs. T2) using linear mixed models as implemented on the GAMLSS software. To identify taxa and functional pathways associated with BMI and/or changes between T1 and T2, we used linear mixed models implemented on the GAMLSS software package for R (3, 4). In particular, we used the following regression formulas to test the association with BMI: “`abundance ~ 1 + Gender + Age + BMI +re(random=~1|User.id, method = ‘REML’)`”, and Time Point: “`abundance ~ 1 + Gender + Age + Time.point +re(random=~1|User.id, method = ‘REML’)`” with the following options ‘`gamlss(...., control=gamlss.control(c.crit = 0.001, n.cyc = 200), family = BE())`’, where “abundance” corresponds to the rarified counts of the taxa or functions divided by the total counts of the sample and “BE()” is the beta distribution family. We performed corrections for multiple hypothesis tests using the local FDR methodology implemented on the “ashr” software package for R (5).

We tested the association between the network module’s eigenvector and BMI and time point using linear mixed models implemented on the lmerTest software package for R [6]. The BMI model used the following options “`lmerTest::lmer(Module_EigenVector ~ 1 + BMI + Gender + Age + (1|User.id), data=pheno_me))`” for the Time Point model we replaced BMI for time point in the formula. We identified modules that were associated with either BMI or time point and those that are associated with both.

Results were corrected for multi-testing using Sidak's methodology, which calculates the family-wise corrected p-values $p^{Sidak} = 1 - (1 - p)^k$ where $p$ is the univariate p-value and $k$ = 3 is the number of hypotheses tested.

## References


1. Langfelder, P, Horvath, S. WGCNA: an R package for weighted correlation network analysis. *BMC Bioinformatics*. (2008) 9(1):559. doi:10.1186/1471-2105-9-559
2. Zhang, B, Horvath, S. A General Framework for Weighted Gene Co-Expression Network Analysis. *Stat Appl Genet Mol Biol*. (2005) 4(1). doi:10.2202/1544-6115.1128
3. Rigby, RA, Stasinopoulos, DM. Generalized additive models for location, scale and shape (with discussion). *J R Stat Soc Ser C Appl Stat*. (2005) 54(3):507-554. doi:10.1111/j.1467-9876.2005.00510.x
4. Stasinopoulos, M, Rigby, R. gamlss.dist: Distributions for Generalized Additive Models for Location Scale and Shape. Published online (2022) https://CRAN.R-project.org/package=gamlss.dist
5. Stephens, M. False discovery rates: a new deal. *Biostatistics*. Published online October 17, 2016 (2016) kxw041. doi:10.1093/biostatistics/kxw041
6. Kuznetsova, A, Brockhoff, PB, Christensen, RHB. lmerTest Package: Tests in Linear Mixed Effects Models. *J Stat Softw*. (2017) 82(13). doi:10.18637/jss.v082.i13
